# Dependence of the Surface Morphology and Micromechanical and Sclerometric Properties of Al_2_O_3_ Layers on the Parameters of Anodizing Aluminum Alloy

**DOI:** 10.3390/ma15238482

**Published:** 2022-11-28

**Authors:** Mateusz Niedźwiedź, Marek Bara, Adrian Barylski

**Affiliations:** Institute of Materials Engineering, Faculty of Science and Technology, University of Silesia in Katowice, 41-500 Chorzów, Poland

**Keywords:** aluminum alloys, oxide layers, microhardness, adhesion, surface morphology

## Abstract

The article presents the dependence of the morphology as well as micromechanical and sclerometric properties of Al_2_O_3_ layers on the parameters of anodizing of aluminum alloys. The oxide layers were produced on the EN AW-5251 aluminum alloy by means of a direct current anodizing in a three-component electrolyte. The input variables (current density and electrolyte temperature) were selected based on the overall design of the experiment. The current density was 1, 2, 3 A/dm^2^, and the electrolyte temperature was 283, 293, 303 K. The surface morphology was examined using a scanning electron microscope (SEM), and then the microscopic images were analyzed using a graphics program. The micromechanical and sclerometric properties were examined by determining the *H_IT_* hardness and three critical loads: *Lc1* (critical load at which the first damage of the tested layers occurred-Hertz tensile cracks inside the crack), *Lc2* (critical load at which the first cohesive damage of the layers occurred) and *Lc3* (load at which the layers were completely damaged). Sclerometric tests with the use of scratch tests were supplemented with pictures from a scanning microscope, showing the scratches. The produced layers are characterized by a hardness above 3 GPa and a porosity of 4.9–10.3%. Such a range of porosity of the produced layers allows their wide application, both for sliding associations with polymers and for their modification.

## 1. Introduction

In recent years, a very large increase in the use of aluminum alloys as a construction material in the broadly understood industry can be observed, in such areas as aviation, machinery, electronics, the automotive and construction industries, and also in nanotechnology (production of membranes for sensors and nanowires) [1,2,3]. Aluminum is gaining more and more popularity due to a number of favorable properties: high strength-to-weight ratio, ease of production, high ductility, light thermal conductivity and high corrosion resistance [4,5]. It should also be noted that aluminum is almost 100% recyclable without losing its properties [6]. In order to increase the mechanical strength and corrosion protection on the surface of aluminum alloys, so-called oxide layers are produced. The most common method of producing Al_2_O_3_ layers is an electrochemical process called anodizing (oxidation) and hard anodizing [7,8]. An important element of the electrochemical anodic oxidation process is the formation of the Al_2_O_3_ layer at the expense of the loss of the aluminum alloy substrate. Due to the above, the oxide layer obtains very high adhesion to the substrate, and can only be separated from the substrate in a mechanical way, involving damage to both the layer and the substrate [9,10]. The oxide layer consists of two layers. The first (inner) layer is called the barrier layer (up to hundreds of nanometers thick). The second layer with a porous surface is the proper layer, up to several hundred micrometers thick. The proper layer has a cellular structure resulting from the dissolving effect of the electrolyte in the pore base [11]. On the surface of the Al_2_O_3_ layer there are micropores, nanopores, and defects and distortions of the oxide layer. The formation of micropores is conditioned by disturbances in the structure as a result of the transfer of substrate defects to the surface of the oxide layer [12]. The formation of nanopores takes place by dissolving the oxide in the barrier layer as a result of the flow of current influencing the increase in the temperature of the electrolyte. A nanopore cell forms hexagonal channels by linear contact with the six surrounding fibers [13]. The type, shape and size of nanopores in the oxide layer largely depend on the parameters of the anodizing process and the manufacturing method used. The structure of the substrate and the etching conditions of the aluminum alloy surface before anodizing also have a great influence [14,15]. The porosity and size of the Al_2_O_3_ nanopores affect their application and properties. Therefore, in order to determine the number and size of nanopores, surface tests are carried out using a scanning microscope, and then image analysis is used [16]. An important feature of anodic aluminum oxide layers is their increase in hardness and abrasion resistance in relation to the aluminum alloy. This contributes to the wide use of oxide layers in sliding associations in engineering kinematic systems. The surface porosity has a significant influence on the application of the oxide layers. The pores between the aluminum oxide fibers can also form the matrix for the dispersion phase in the production of composite surface layers on a substrate of aluminum alloys. In this case, higher porosity oxide layers are used. For this reason, tests are often carried out to verify the influence of anodizing parameters and porosity on the mechanical and sclerometric properties of oxide layers. In order to determine the abrasive wear resistance of the oxide layers, short-term sclerometric tests are performed [17,18,19].

The analysis of the literature shows that the vast majority of scientific research for various applications concerns oxide layers produced under a constant electric charge [20,21,22,23]. There were no publications concerning the influence of anodizing parameters (electrolyte temperature, current density) on their properties as a result of constant-time anodizing, which was the aim of the presented research.

## 2. Materials and Methods

### 2.1. Research Material

The base material used in the research was the aluminum alloy EN AW-5251 (produced by ZML in Kęty). This alloy was chosen due to its good mechanical properties, corrosion resistance and low content of impurities of other elements, which is advantageous for its surface anodizing. The chemical composition of the aluminum alloy EN AW-5251 is presented in Table 1. Samples with dimensions of 62.5 × 16 mm were cut from a sheet of 4 mm thick using water-jet cutting technology. In the next step, the samples were ground using a SAPHIR 520 single-disc grinding and polishing machine in order to even out the edges. A hole was made on the side surface of the samples, which was then threaded. Threaded rods made of the same aluminum alloy as the samples were screwed into the thread previously made in the sample. The rods served as an element to connect the sample (anode) with the electrical circuit.

**Figure 1:**
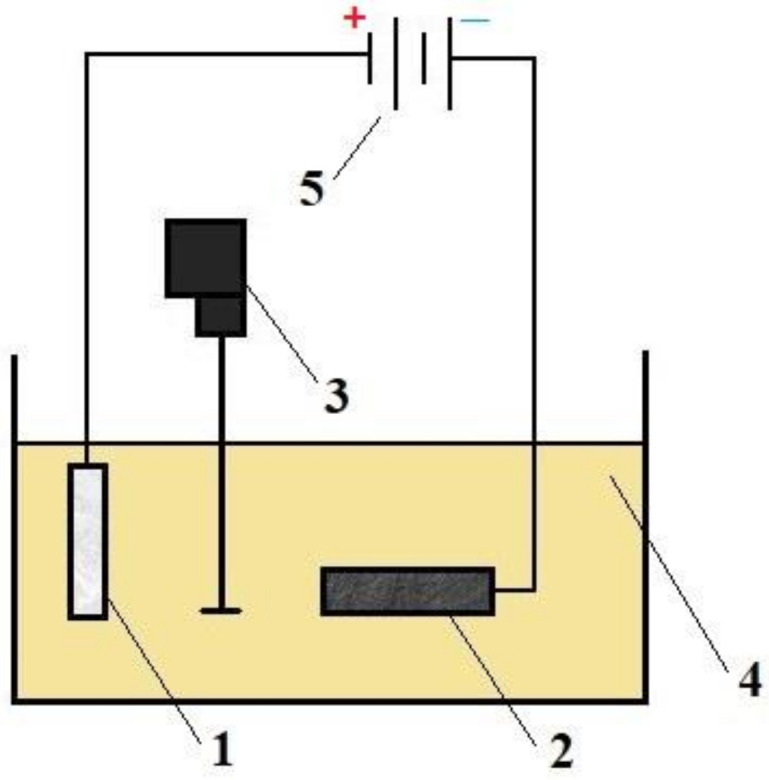
Diagram of the anodizing system used during the production of the oxide layer: (**1**) aluminum alloy EN AW-5251 (anode); (**2**) lead plate (cathode); (**3**) mechanical stirrer; (**4**) electrolyte; (**5**) power supply.

The surfaces of the samples not participating in the tests, together with the rod, were glued with electrolyte-resistant glue in order to limit the anodized surface, and thus limit the anodizing current value in the electrochemical process. The surface of aluminum and its alloys in contact with the atmosphere was covered with a thin layer of alumina passivation, and therefore the surfaces to be anodized could be etched immediately before the electrochemical process. The samples were digested in a 5% KOH solution for 20 min and then neutralized in a 10% HNO_3_ solution to reverse the digestion reaction. The treatments ended with rinsing in distilled water. The electrochemical process was carried out in an electrolyte consisting of an aqueous solution of acids: 18% H_2_SO_4_ (33 mL/l), C_2_H_2_O_4_ (30 g/l) and C_8_H_6_O_4_ (76 g/l). The addition of C_8_H_6_O_4_ acid ensures obtaining hard layers and enables the process to be carried out at room temperature. The cathode in the electrochemical process was a lead plate with dimensions corresponding to the dimensions of the samples (anode). During the anodizing process, the electrolyte was mechanically stirred at a speed of 100 rpm. Cooling the electrolyte during the anodizing process is necessary due to the release of a significant amount of heat in the area of layer growth, which accelerates the hydration process and inhibits the growth of the layer. The diagram of the anodizing process is shown in Figure 1.

The anodizing parameters were selected on the basis of the experimental plan. Table 2 shows the total experimental plan for two input fields with three variable values.

The input variables were the current density 1, 2, 3 A/dm^2^ and the electrolyte temperature 283, 293, 303 K. The anodizing time for all samples was 20 min. Anodizing was performed using the DC method using a stabilized GPR-25H30D power supply. After the anodizing process was completed, the samples were rinsed in distilled water for 60 min.

### 2.2. Research Methodology

**Figure 2:**
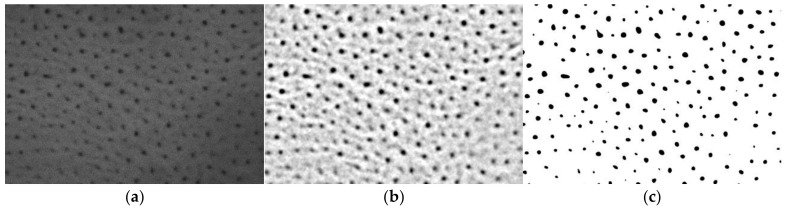
Stages of preparing SEM images for stereological analysis: (**a**) noise reduction in the image; (**b**) filtering the image in a band-like manner; (**c**) binarization.

Microscopic examinations were performed using a Hitachi S-4700 scanning electron microscope (Hitachi, Tokyo, Japan). Surface morphology images for nanoporosity analysis were taken at a magnification of 50,000×. The microscopic observations of the scratches made after the sclerometric tests were made at 35× magnification. Aluminum oxide layers are poorly conductive, so they charge electrically when the electron beam is operated, which contributes to erroneous observations. For proper observation, the layers were sprayed with carbon using a carbon sputtering machine. The carbon layer makes it possible to discharge the bouncing electrons during the tests, enabling proper observation. ImageJ 1.50i (Wayne Rasband, MD, USA) was used for computer analysis of SEM digital microscopic images. SEM images of the surface of the oxide layers with dimensions of 2520 × 1753 nm were examined. The analysis was preceded by the appropriate preparation of the images by noise reduction with the Smooth tool, band filtering with the Bandpass Filter tool and binarization with the Thresholding tool. The individual stages of preparing images for analysis are shown in Figure 2.

Using appropriate computer image analysis procedures, mean values of stereological surface parameters were determined. The research allowed us to determine the porosity of the surface of the layers, the number of nanopores per unit of surface area and the size of the nanopores. The data was used to make histograms showing the surface area distribution of the nanopores.

The thickness of the oxide layers was measured by the contact method using a Fischer Dualscope MP40 instrument (Helmut Fischer GmbH + Co., KG, Shin-delfingen, Sindelfingen, Germany). This device takes measurements using the eddy current method. On the surface of each sample, 10 measurements were made, which were then used to calculate the average value of the thickness of the oxide layer with standard deviations.

Micromechanical tests were performed using the Micro Combi Tester-MCT3 (Anton Paar, Corcelles-Cormondrèche, Switzerland). A Berkovich diamond indenter (B-V 83) was used. A maximum load of 50 mN was applied, and the loading and relieving of the indenter were performed at a speed of 100 mN/min. The holding time under the maximum load was 10 s. Six impressions were made for each sample. The *H_IT_* hardness was determined by the Oliver–Pharr method [24]. Measurements were made in accordance with the recommendations of ISO 14577 [25].

The sclerometric tests were performed during the standard scratch test of the surface of the layers with increasing load force on the Micro Combi Tester-MCT3 by Anton-Paar (Corcelles-Cormondrèche, Switzerland). The tests were carried out in accordance with the guidelines of ISO 20502 [26] and ASTM C1624 [27] using a 100 μm Rockwell diamond indenter. Each test was divided into 3 stages. In the first (pre-scan), the profile of the sample was scanned under a load of 0.03 N. In the second stage (scan) the appropriate tests were performed with a progressively increasing load from 0.03 to 30 N. The length of each scratch was 3 mm. The speed of the indenter movement was 6 mm/min. The last stage (post-scan) consisted in scanning the profile under a load of 0.03 N resulting from scratching the surface. Parameters such as pressure force *Fn* [N], penetration depth of the indenter under load *Pd* [μm] and depth of penetration after unloading *Rd* [μm] were recorded.

Photos of the surface show three critical loads: *Lc1* (critical load at which the tested layer was damaged-Hertz cracks extending inside the scratch), *Lc2* (critical load at which cohesive failure of the layer occurred) and *Lc3* (load at which the layer was completely damaged).

## 3. Results and Discussion

### 3.1. Morphology and Image Analysis

**Figure 3:**
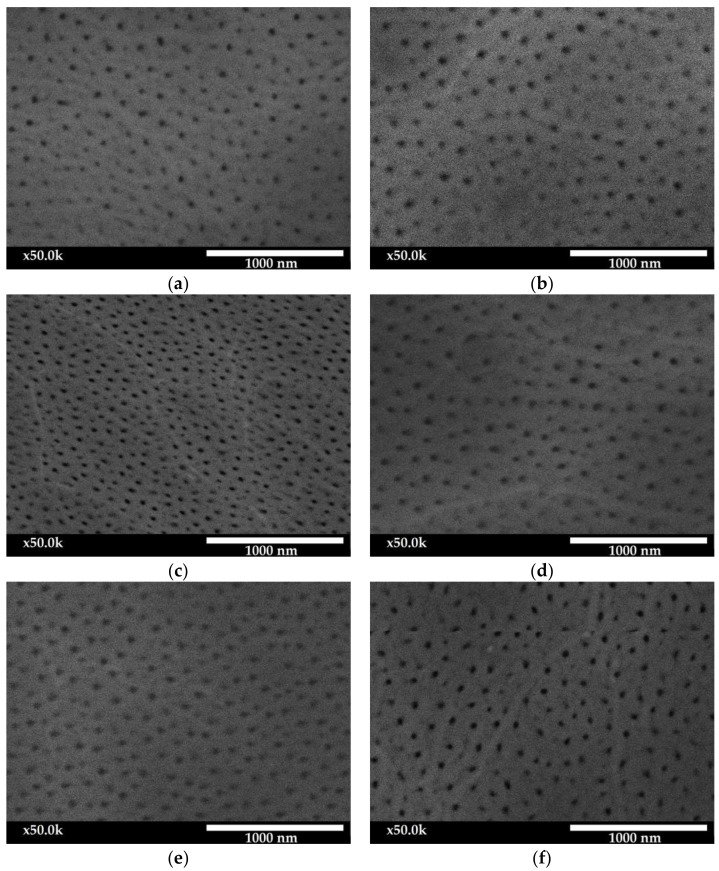

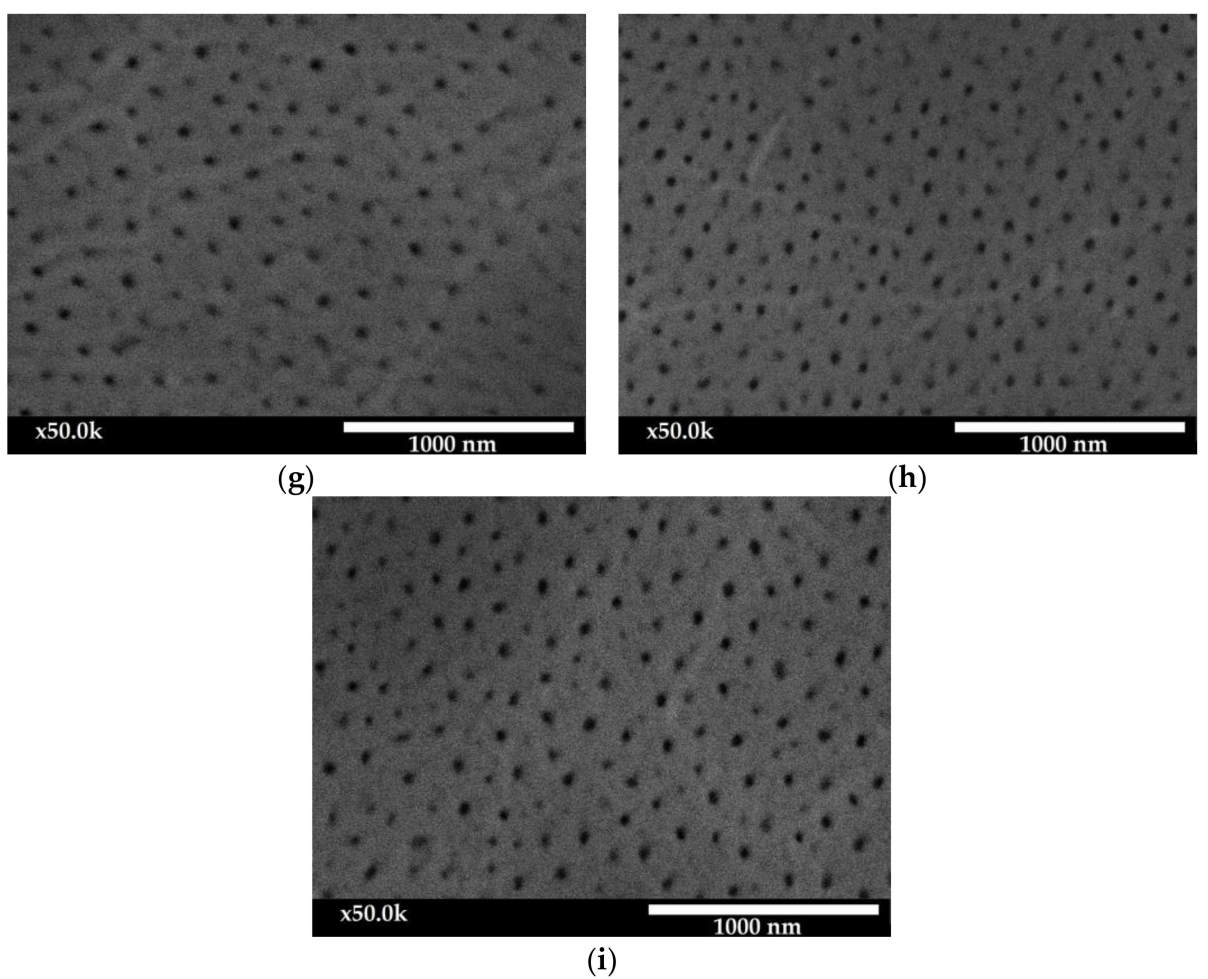
Images of surface morphology of oxide layer: (**a**) Sample A; (**b**) Sample B; (**c**) Sample C; (**d**) Sample D; (**e**) Sample E; (**f**) Sample F; (**g**) Sample G; (**h**) Sample H; (**i**) Sample I.

With the use of scanning electron microscopy (SEM), it is possible to observe the porosity of the oxide layers, which is the basic property of aluminum oxides. Figure 3 shows the SEM images for the surface of the Al_2_O_3_ layers at a magnification of 50,000×.

The surface morphology images show the nanoporosity characteristic of oxide layers, which is a channel for the migration of oxygen ions connecting with the anode material in the electrical and chemical processes.

**Figure 4:**
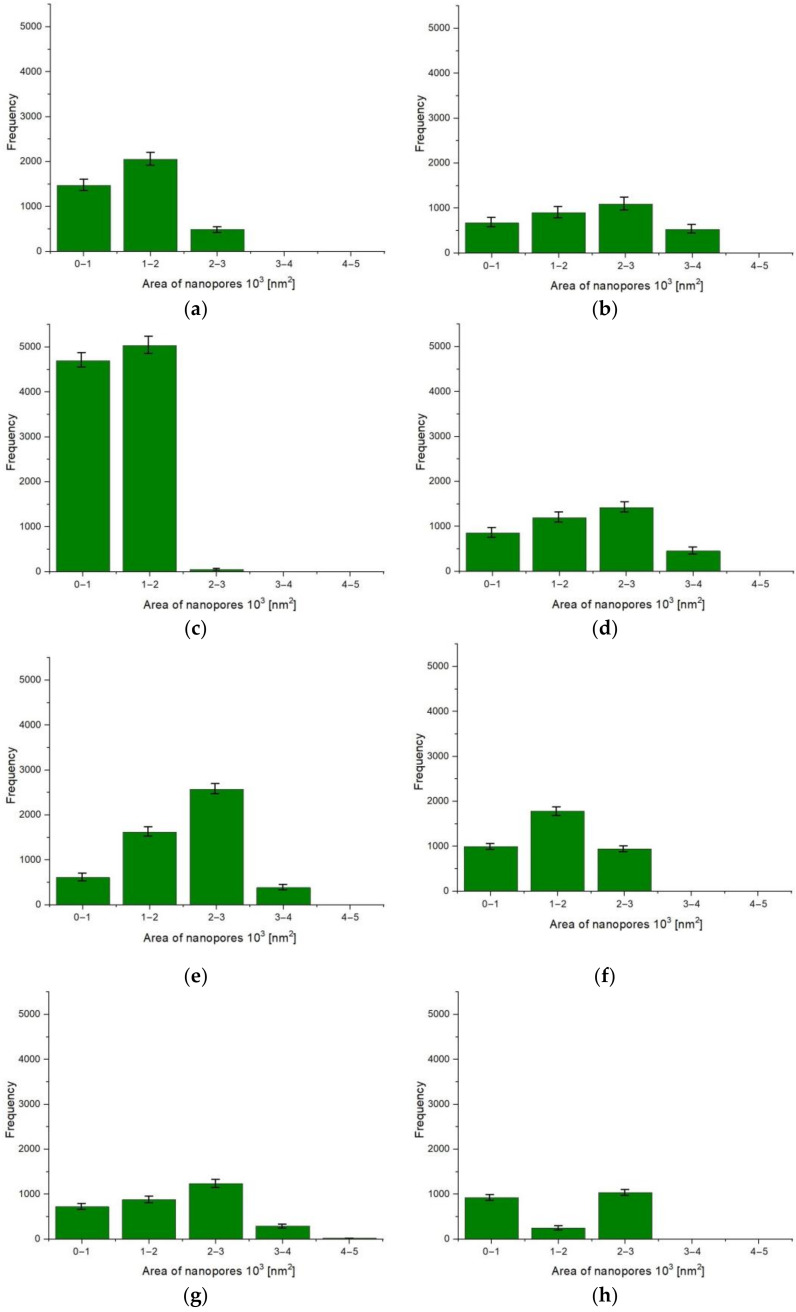

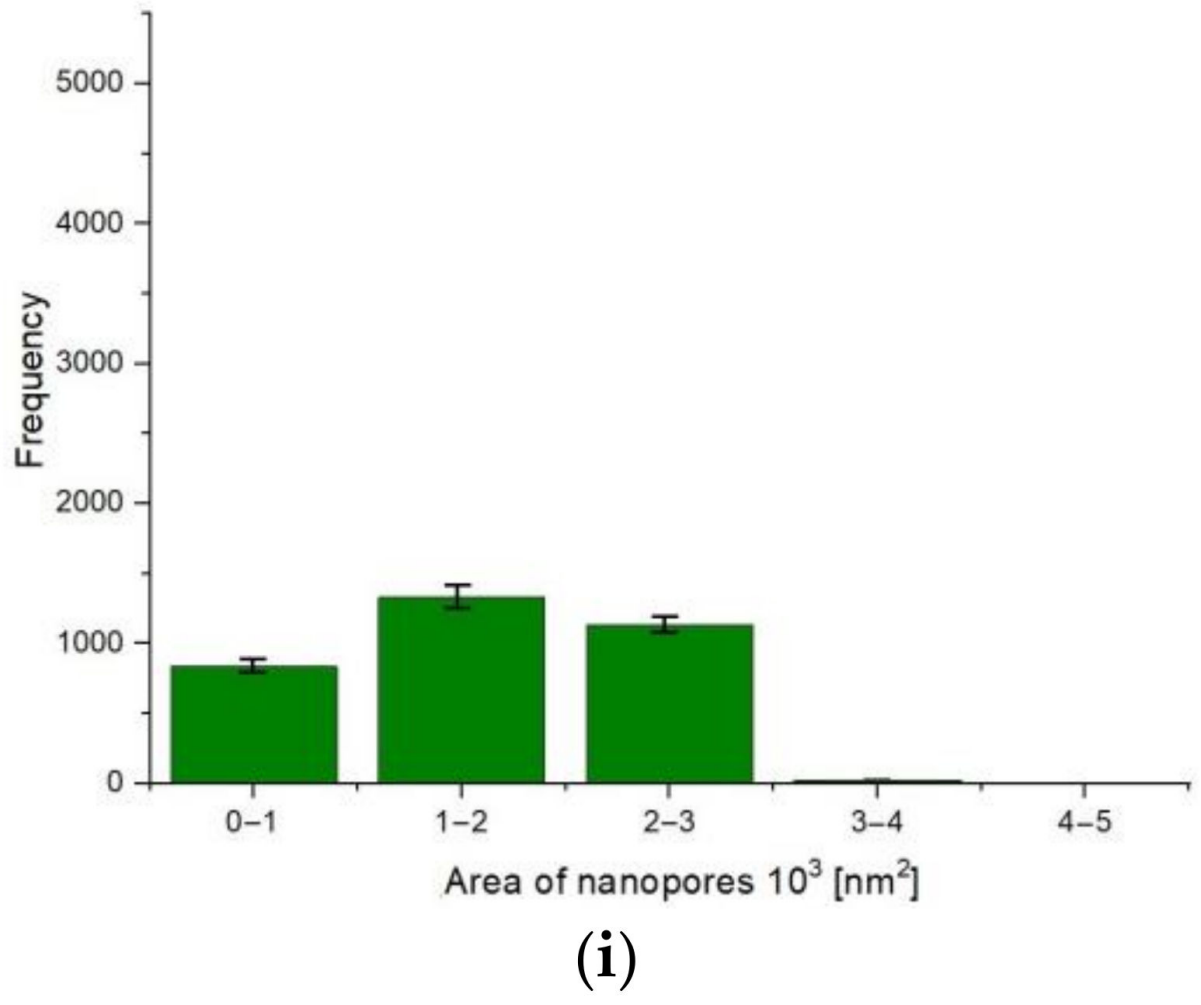
Histogram of occurrence of nanopores and their surfaces with standard deviations: (**a**) Sample A; (**b**) Sample B; (**c**) Sample C; (**d**) Sample D; (**e**) Sample E; (**f**) Sample F; (**g**) Sample G; (**h**) Sample H; (**i**) Sample I.

Based on the image analysis, the results of the stereological parameters were obtained (Table 3), which were used to prepare histograms of the distribution of the surface of nanopores in individual layers (Figure 4). In order to better visualize the distribution of nanopores on the surface of the Al_2_O_3_ layer, the values from the table were converted for the area of 100 µm^2^. All image analysis calculations were made on the basis of three images of the layer from different locations.

The layer of sample E produced at a current density of 1 A/dm^2^ and an electrolyte temperature of 293 K is characterized by the highest porosity and the highest average nanopore area. The lowest porosity was determined for the anodized oxide layer at the lowest production parameters: current density 1 A/dm^2^ and electrolyte temperature 283 K (Sample A). The Al_2_O_3_ layer of sample C produced at a current density of 1 A/dm^2^ and a temperature of 303 K is characterized by the smallest average nanopore field and the highest nanopore density per µm^2^.

Significant changes in the surface morphology of Al_2_O_3_ layers, resulting from the applied conditions of the anodizing process, were observed. The increase in the current density at an electrolyte temperature of 283 K causes a linear increase in both the porosity and the average surface area of the nanopores. The histograms also show an increase in the proportion of pores with a larger surface area. The layers produced in the electrolyte with a temperature above 283 K at a constant current density of 1 A/dm^2^ showed an approximately two-fold increase in porosity. The layer produced at a current density of 1 A/dm^2^ with an electrolyte temperature of 303 K is characterized by a very high pore density of up to 98 pores/µm^2^; however, these are pores with very small areas (up to 2000 nm^2^). The histograms also show a high proportion of pores with large surfaces for samples E and B; the vast majority of pores have an area of 2000–3000 nm^2^, and a significant number of pores with an area of over 3000 nm^2^ is also noticeable.

### 3.2. The Thickness of the Oxide Layer

Table 4 shows the average values of the thickness measurements of the oxide layers with standard deviations.

**Figure 5:**
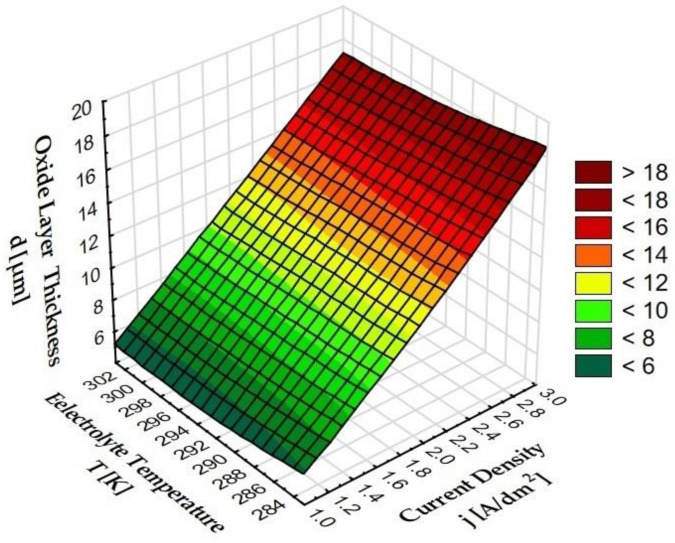
Dependence of the thickness of oxide layers on the current density and electrolyte temperature.

Figure 5 shows the dependence of the parameters of oxide layer production (current density, electrolyte temperature) on the thickness of these layers.

**Figure 6:**
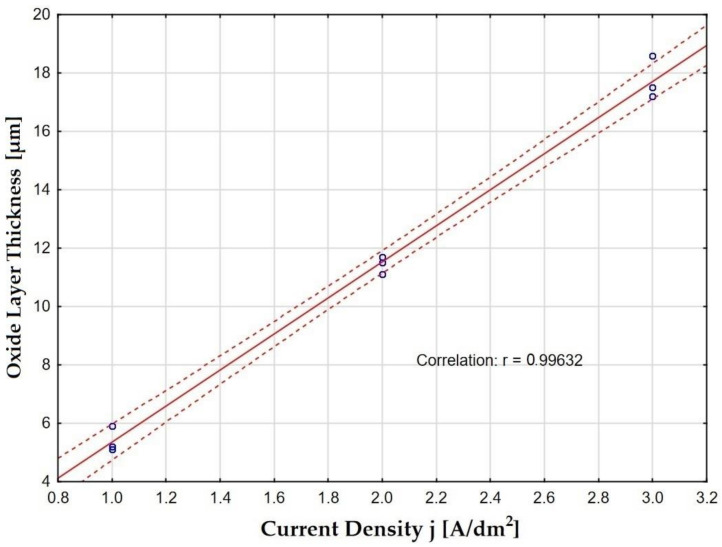
Effect of current density on layer thickness, along with the confidence interval.

As a result of the thickness measurements carried out, it can be concluded that the parameters of the electrochemical process (anodizing current density) have a significant impact on the thickness of Al_2_O_3_ layers in a constant process time (Figure 6). A significant increase in the thickness of the layer was observed when the anodizing current density increased at a constant electrolyte temperature. This is due to the increase in the value of the electric charge resulting from the increase in the anodizing current at a constant value of the anodized surface. The increase in the temperature of the electrolyte, at a constant current density, causes a decrease in the thickness of the layer, which results from the increase in solubility of the secondary Al_2_O_3_ layer by the electrolyte at a higher temperature. Sample B, with the largest thickness (18.6 μm), is a layer produced at a current density of 3 A/dm^2^ in an electrolyte at a temperature of 283 K. The smallest layer thickness (5.1 μm) was shown for Sample C, on which a layer was produced at the density current of 1 A/dm^2^ in the electrolyte at a temperature of 303 K.

### 3.3. Influence of Anodizing Parameters on Micromechanical Properties

The undoubted advantage of anodic oxide layers produced in the electrochemical process is the ability to control their operational properties by changing their porosity (Table 3), thickness (Table 4) and microhardness (Table 5), as a result of the selection of the conditions for the production of layers (electrolyte temperature, current density). Table 5 shows the average values of the microhardness of *H_IT_* oxide layers produced with various parameters according to the overall plan, with standard deviations. The maximum penetration depth h is also included, which facilitates the interpretation of microhardness changes.

**Figure 7:**
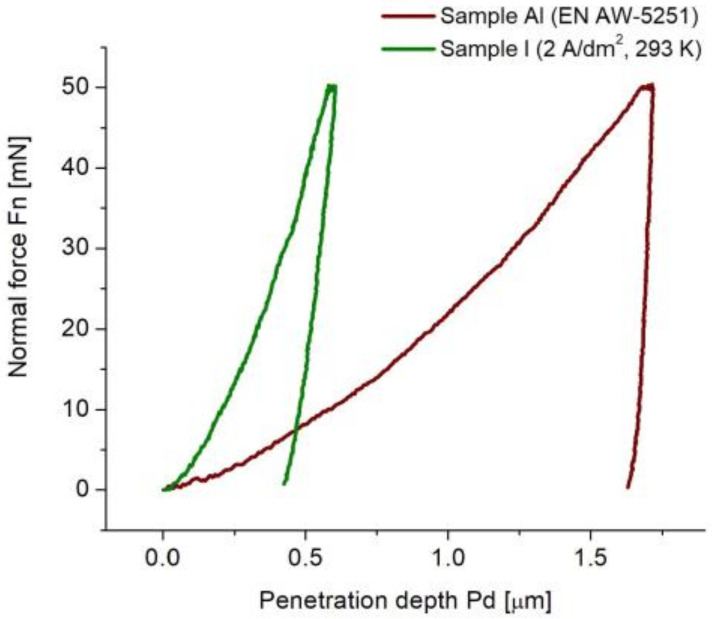
Load–unload curves for: aluminum alloy surface (Simple Al) and oxide layer (Sample I).

The oxide layers produced in order to improve the abrasion resistance of the surface of aluminum alloys (reducing the friction coefficient and the wear intensity of triboelements) are produced by the so-called hard anodizing method. Hard anodizing is considered to be the process of anodic production of oxide layers on aluminum and its alloys by an electrochemical method, as a result of which the oxide layer obtains a microhardness of at least 3 GPa. As a result of the research analysis, it can be concluded that the electrochemical process is a hard anodizing process, because all the produced layers are characterized by a hardness above 3 GPa. The oxide layers obtained on the EN AW-5251 alloy with the electrochemical method are mainly characterized by an increase in hardness compared with the parent metal (see Table 5 for a comparison of the Al sample with other samples). The increase in hardness ranges from 4.5 times for the layer with the lowest hardness to 8.5 times for the hardest layer. The increase in hardness as a result of the electrochemical process can also be observed in the graphs of the load–unload curves, where there is a significant difference in the penetration depth of the indenter for the surface of the aluminum alloy and oxide layers (Figure 7).

**Figure 8:**
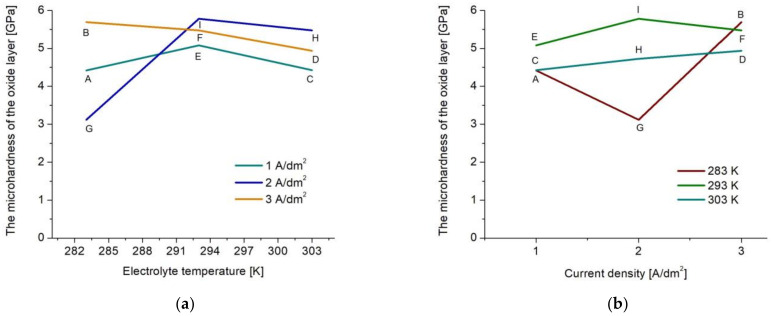
Dependence of the microhardness of oxide layers on changes in: (**a**) current density; (**b**) electrolyte temperature.

Due to the increase in surface hardness, and thus the tribological wear resistance, it is possible to successfully use aluminum elements with layers produced on them as parts in kinematic sliding nodes of machines and devices. The changes in the microhardness of the oxide layers depending on the production parameters show a form of a function close to linear only for the layers produced at the temperature of 303 K and the density of 3 A/dm^2^ (Figure 8). For a constant current density of 3 A/dm^2^, the increase in temperature causes a decrease in microhardness (Figure 8a). For a constant 303 K electrolyte temperature, an increase in the current density causes an increase in the microhardness (Figure 8b). However, these changes are not significant. In other cases, the characteristics take the form of parabolic curves.

The highest values of microhardness were demonstrated for layers produced at a current density of 3 A/dm^2^ in the electrolyte at a temperature of 283 and 303 K (samples B, F) and for sample I produced at a current density of 2 A/dm^2^ in an electrolyte at a temperature of 293 K. A current density of 1 A/dm^2^ resulted in obtaining layers with lower microhardness (samples A, C). In the case of changes in the electrolyte temperature’s tested ranges, the greatest changes in the microhardness occur for the current density of 2 A/dm^2^ (sample G, I), whereas in the case of changes in the current density, the greatest changes in the microhardness occur for the temperature of 283 K (sample G, B). The layers produced at 283 and 303 K show lower microhardness values, whereas the layer produced at 293 K shows high microhardness. To sum up, in order to increase the microhardness of oxide coatings obtained on aluminum alloys, the anodizing process should be carried out at a current density of 3 A/dm^2^ or at a temperature of 293 K.

In many scientific works, oxide layers produced on aluminum alloys are modified with solid lubricants in the form of e.g., graphite, molybdenum disulphide or tungsten disulphide, or are used for anti-corrosion protection, improving aesthetics (colored layers) or electrical insulation. The increase in the content of the composite component in the structure of the oxide layer is very often influenced by high porosity or large diameters of nanopores. For this reason, it is advantageous to optimize the conditions for producing the oxide layer so as to obtain high porosity while maintaining high microhardness. The conducted research shows that the best parameters for the production of oxide layers in order to obtain a matrix for composite layers are the current density of 1 A/dm^2^ and the electrolyte temperature of 293 (sample E), with which were produced a high microhardness of 5.077 GPa and very high porosity—as much as 10.3%.

### 3.4. Adhesive Properties of Anodic Oxide Layers

The advantages of oxide layers produced on aluminum alloys also include their excellent adhesion to the substrate. The most common method of testing the adhesion forces of a layer to the substrate is the scratch test. As a result of such a test, scratches on the surface of the layer are obtained, which may have the character of Hertz cracks occurring usually at the beginning of loading the layer (cohesive damage), and adhesive cracks, which completely damage layers. Cohesive cracks result from the action of forces inside the torn layer and appear as cracks in a direction perpendicular to the direction of movement of the indenter. Adhesive cracks result from the action of significant compressive stresses arising in the layer and are revealed by the crushing and tearing of the layer in front of the indenter.

**Figure 9:**
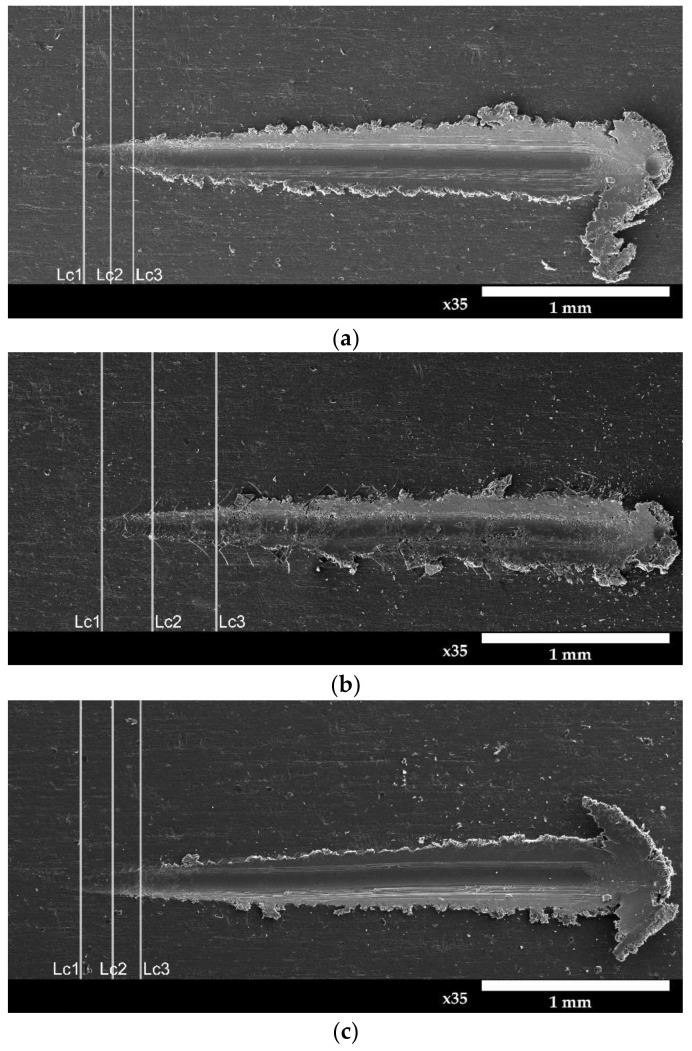

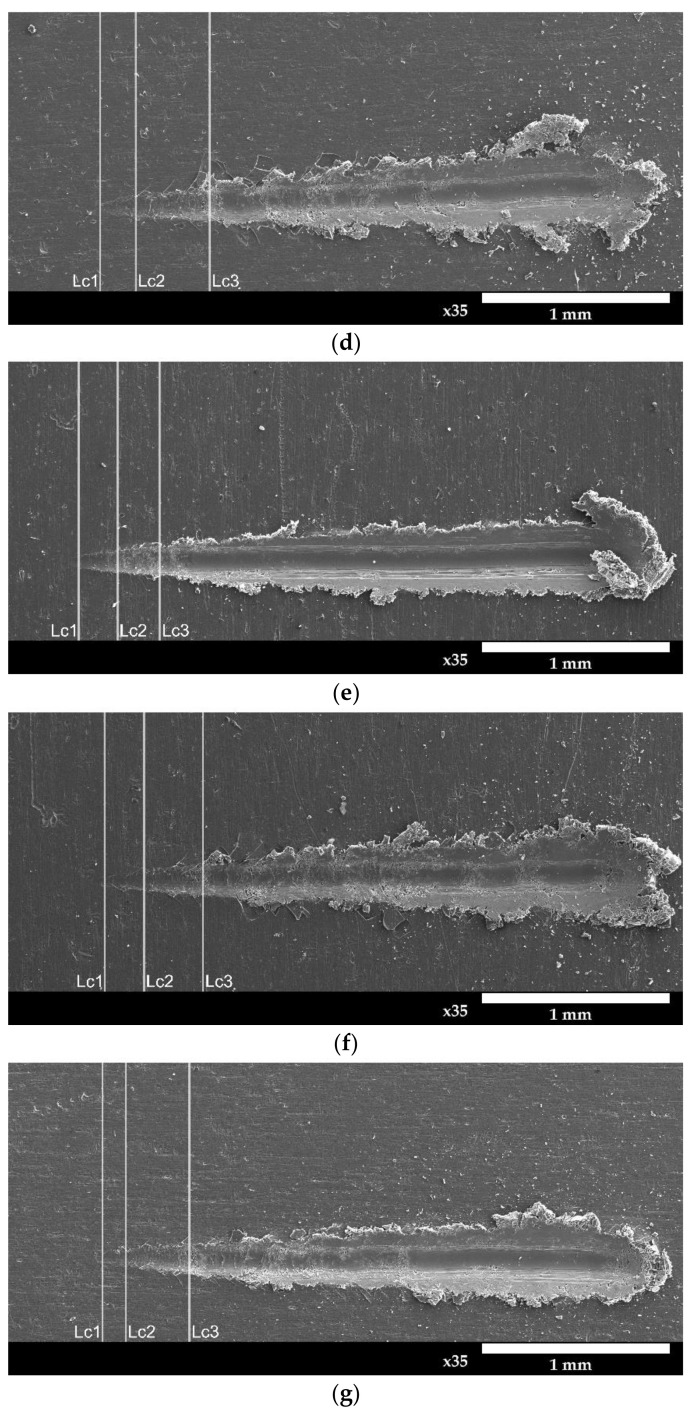

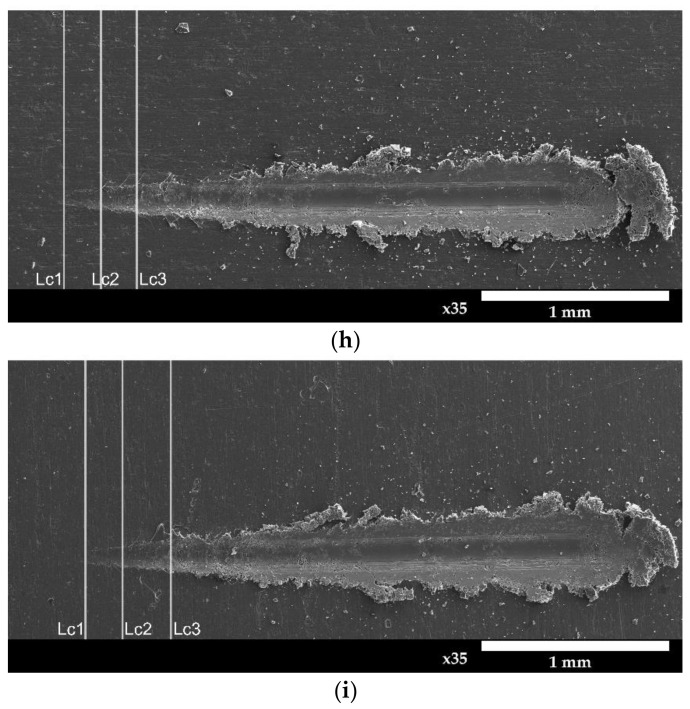
Critical loads determined for layers: (**a**) Sample A; (**b**) Sample B (**c**) Sample C; (**d**) Sample D; (**e**) Sample E; (**f**) Sample F; (**g**) Sample G; (**h**) Sample H; (**i**) Sample I; *Lc1*—Hertz cracks; *Lc2*—cohesive cracks; *Lc3*—adhesive cracks and complete damage of layer.

The following phenomena are visible on the samples subjected to the scratch tests: plastic deformation, circular Hertz cracks, forward V-shaped cracks (at the edges inside and outside of the scratch), deformed coform cracks and cohesive chipping along the scratch marks. The places where the Hertz-type cracks occurred, as well as the cohesive and adhesive cracks, are shown in Figure 9 with the critical loads marked. The mean values of the critical loads *Lc1*, *Lc2* and *Lc3* are presented in Table 6.

**Figure 10:**
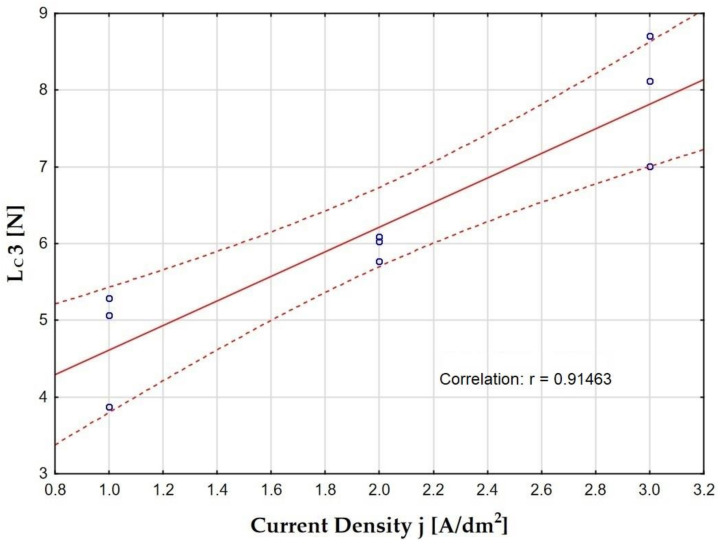
Effect of current density on Lc3, along with the confidence interval.

The analysis of the adhesion test results showed that the scratch resistance of the layers largely depends on the current density (Figure 10); therefore it correlates more with the thickness of the produced layers than with their hardness.

The layers, produced at a current density of 3 A/dm^2^, whose thicknesses are the largest (samples B, D, F), show the highest mechanical strength (the highest values of the *Lc3* parameter). On the other hand, the layers produced at a current density of 1 A/dm^2^, the thicknesses of which are the smallest (samples A, C, E), have the lowest mechanical strength (the lowest values of the *Lc3* parameter). The lowest scratch resistance was demonstrated by the layer produced at the current density of 1 A/dm^2^ in the electrolyte at 283 K. For this layer, the first damage was observed at the load of *Lc1* = 1.43 N. The layer was completely broken at the load of *Lc3* = 3.87 N. The layer produced at a current density of 3 A/dm^2^ in an electrolyte at a temperature of 303 K showed the highest mechanical strength. Load *Lc3* is equal to 8.71 N (which is more than a double increase compared with the layer with the lowest strength). The layer produced at the current density of 3 A/dm^2^ in the electrolyte at the temperature of 293 K also showed significant mechanical strength. For this layer, the highest load values were observed for the Hertz cracks of 2.42 N and for cohesive cracks of 4.29, and the complete tear of the layer occurred at a load of 8.12, which is slightly less than that for the layer produced at the same current density but in an electrolyte with a temperature of 303 K.

## 4. Conclusions

The research results and their analysis presented in the article confirm the sense of modifying the surface of the EN AW-5251 aluminum alloy in the electrochemical process in order to increase the hardness and strength of its surface. The three-component electrolyte used in the tests enables the formation of hard oxide layers at room temperature (283–303 K) without the need for intensive cooling, as is the case with anodizing in acid solutions (COOH)_2_ or H_2_SO_4_, which significantly reduces costs of the conducted process. The highest values of microhardness were demonstrated for layers produced at a current density of 3 A/dm^2^ in the electrolyte at 283 and 303 K and at a current density of 2 A/dm^2^ in the electrolyte at 293 K. The scratch resistance of the layers correlates more closely with their thickness than with their hardness. As a result of strength tests, cracks are obtained on the surface of the layer, which may be elastic Hertzian cracks inside the layer (Lc1), cohesive cracks (Lc2) or adhesive cracks (Lc3). As a result of the action, cohesive cracks are formed by forces inside the broken layer and appear in the form of cracks in the direction perpendicular to the direction of movement of the indenter. Adhesive cracks result from the action of significant compressive stresses arising in the layer and revealing themselves as a result of crushing and tearing beyond the layer before the indenter. The layers produced at a current density of 3 A/dm^2^, the thicknesses of which are the highest, show the highest mechanical strength. All the produced layers are characterized by a hardness above 3 GPa, and thus they can be used in sliding kinematic nodes. The anodizing parameters used in the research make it possible to obtain layers with a porosity of 4.9–10.3%. Such a range of porosity of the produced layers allows their wide application, both for sliding associations with polymers and for their modification. The highest values of porosity were demonstrated for the layer produced at the current density of 1 A/dm^2^ in the electrolyte at the temperature of 293 K. The greatest correlations were shown for the dependence of layer thickness and mechanical strength on the current density.

## Figures and Tables

**Table 1:** Chemical composition of EN AW-5251 aluminum alloy.

Fe	Si	Zn	Ti	Mg	Mn	Cu	Cr	Other	Al
Max	Max	Max	Max	1.7	0.1	Max	Max	Max	Min
0.5	0.4	0.15	0.15	2.4	0.5	0.15	0.15	0.15	rest

**Table 2:** Total experiment plan.

Sample	Controlled Factors			
	On a Natural Scale		On a Standard Scale	
	Current Density j [A/dm^2^]	Electrolyte Temperature T [K]	×1	×2
A	1	283	−1	−1
B	3	283	1	−1
C	1	303	−1	1
D	3	303	1	1
E	1	293	−1	0
F	3	293	1	0
G	2	283	0	−1
H	2	303	0	1
I	2	293	0	0

**Table 3:** Values of stereological parameters from computer image analysis.

Sample	Porosity(%)	Standard Deviation (%)	Pore Density (Number of Pores/µm^2^)	Standard Deviation (Number of Pores/µm^2^)	Average Area of Nanopores (nm^2^)	Standard Deviation (nm^2^)
A	4.9	0.08	40.1	1.8	1247.7	41.6
B	6.2	0.12	31.8	1.5	1952.2	67.4
C	9.5	0.18	97.7	3.2	977.3	54.2
D	7.3	0.16	39.2	2.1	1870.9	51.6
E	10.3	0.24	51.8	2.4	1981.4	59.4
F	5.3	0.24	37.2	1.6	1436.7	41.8
G	5.9	0.019	31.5	1.8	1857.7	43.2
H	6.7	0.32	44.6	2.1	1504.1	41.1
I	5.3	0.25	33.1	2.2	1589.5	33.6

**Table 4:** List of Al_2_O_3_ layers thicknesses produced in anodizing process.

Sample	Oxide Layers Thickness d [μm]	Standard Deviation [μm]
A	5.9	0.3
B	18.6	0.4
C	5.1	0.2
D	17.2	0.6
E	5.2	0.2
F	17.5	0.4
G	11.7	0.5
H	11.1	0.3
I	11.5	0.4

**Table 5:** Microhardness of studied layers and maximum indentation depth.

Sample	*H_IT_* [GPa]	Standard Deviation [GPa]	*h* [µm]	Standard Deviation [µm]
Al (EN AW-5251 non-etched)	0.682	0.035	1.715	0.035
A	4.419	0.404	0.701	0.023
B	5.697	0.453	0.601	0.023
C	4.422	0.530	0.694	0.050
D	4.934	1.504	0.668	0.110
E	5.077	0.630	0.655	0.042
F	5.474	2.333	0.655	0.135
G	3.118	0.505	0.830	0.063
H	4.725	0.358	0.667	0.027
I	5.783	0.818	0.613	0.037

**Table 6:** Critical loads determined for the tested layers.

Sample	*Lc1* [N]	Standard Deviation [N]	*Lc2* [N]	Standard Deviation [N]	*Lc3* [N]	Standard Deviation [N]
A	1.43	0.05	2.70	0.21	3.87	0.22
B	1.45	0.25	4.01	0.33	7.01	0.12
C	1.85	0.21	3.51	0.02	5.07	0.43
D	1.81	0.81	3.91	0.12	8.71	0.45
E	1.73	0.11	3.49	0.07	5.29	0.16
F	2.42	0.15	4.29	0.01	8.12	0.89
G	2.00	0.91	3.42	0.86	6.03	0.69
H	1.82	0.34	3.42	0.07	5.77	0.16
I	1.93	0.08	3.32	1.06	6.09	0.43

## Data Availability

The data presented in this study are available on request from the corresponding author. The data are not publicly available due to insufficient space to insert data.
